# Baseline Ejection Fraction as a Modifier of Beta‐Blocker Therapeutic Effects in Post‐Acute Coronary Syndrome Patients With Non‐Reduced Ejection Fraction: A Systematic Review and Meta‐Analysis

**DOI:** 10.1155/cdr/2988999

**Published:** 2026-06-17

**Authors:** Seyed Alireza Mirhosseini, Seyed Amir Sadrzadeh, Davood Semirani-Nezhad, Erta Rajabi, Hanieh Mohammadi, Alireza Shaabanpoor Haghighi, Mohammad Amin Jamali, Reza Moshfeghinia, Maryam Ranjbar, Pouria Azami, Rahul Gupta, Wilbert S. Aronow, Armin Attar

**Affiliations:** ^1^ Cardiovascular Research Center, Shiraz University of Medical Sciences, Shiraz, Iran, sums.ac.ir; ^2^ Department of Cardiovascular Medicine, TAHA Clinical Trial Group, School of Medicine, Shiraz University of Medical Sciences, Shiraz, Iran, sums.ac.ir; ^3^ Student Research Committee, Shiraz University of Medical Sciences, Shiraz, Iran, sums.ac.ir; ^4^ Tehran Heart Center, Cardiovascular Diseases Research Institute, Tehran University of Medical Sciences, Tehran, Iran, tums.ac.ir; ^5^ Faculty of Medicine, Tehran University of Medical Sciences, Tehran, Iran, tums.ac.ir; ^6^ MD-MPH Department, School of Medicine, Shiraz University of Medical Sciences, Shiraz, Iran, sums.ac.ir; ^7^ Department of Cardiology, St. Luke’s University Health Network, Bethlehem, Pennsylvania, USA, slhn.org; ^8^ Department of Medicine, Westchester Medical Center and New York Medical College, Valhalla, New York, USA

**Keywords:** acute coronary syndrome, beta-blocker, mortality, myocardial infarction, preserved ejection fraction

## Abstract

**Background:**

Beta‐blockers are regarded as one of the primary treatment options for acute coronary syndrome (ACS) patients with reduced left ventricular ejection fraction (LVEF). However, there is ongoing debate about their therapeutic efficacy in patients with non‐reduced LVEF (≥ 40%).

**Objective:**

To determine the impact of long‐term beta‐blocker administration on major adverse cardiovascular events (MACE) in ACS patients with LVEF ≥ 40*%*.

**Methods:**

A thorough literature search across four databases was conducted to identify eligible studies comparing beta‐blocker therapy with placebo or no medication in addition to standard ACS pharmacotherapy. The outcomes of interest included all‐cause mortality and MACE (comprising cardiovascular mortality, recurrent myocardial infarction [Re‐MI], stroke, rehospitalization for heart failure, and revascularization). Based on LVEF values, patients were divided into three groups: (1) LVEF > 40*%*, (2) 40*%* < LVEF < 50*%*, and (3) LVEF ≥ 50*%*, and the random‐effects meta‐analysis estimated the risk ratios (RRs), hazard ratios (HRs), and 95% confidence intervals (CIs).

**Results:**

Five randomized controlled trials (RCTs) and 19 observational studies met the inclusion criteria. Within the RCTs, beta‐blocker use was associated with a non‐significant 6% reduction in MACE (RR = 0.94, 95*%*CI = 0.87–1.02, *I* ^2^ = 19.1*%*). The pooled analyses of observational studies showed no significant overall impact of beta‐blocker use on endpoint outcomes. Subgroup analyses revealed a reduction in MACE (HR = 0.75, 95*%*CI = 0.59–0.95, *I* ^2^ = 0*%*) and Re‐MI (HR = 0.45, 95*%*CI = 0.23–0.89) in patients with LVEF 40%–49%; however, these findings were derived primarily from observational data. Patients with LVEF ≥ 50*%* did not appear to benefit from long‐term beta‐blocker use.

**Conclusion:**

Long‐term beta‐blocker use did not show a significant benefit in patients with preserved ejection fraction (LVEF ≥ 50*%*). Observational data suggested a possible reduction in MACE and Re‐MI in the LVEF 40%–49% subgroup; however, this finding requires confirmation in dedicated RCTs. Future larger RCTs are needed to clarify the role of beta‐blockers in this borderline population.

## 1. Introduction

Myocardial infarction (MI), as a main subgroup of acute coronary syndrome (ACS), affects more than 2 million individuals globally each year [1]. The potential benefit of beta‐blockers (BBs) in reducing mortality and morbidity among ACS patients was first suggested in 1965 and gained further support in the 1970s [2]. Over the past decades, BBs have been prescribed for ACS patients in addition to other routine treatments to prevent ongoing ischemic events and related heart failure (HF) based on American and European guidelines [3–5].

Numerous randomized controlled trials (RCTs) and large‐scale observational studies have investigated the efficacy of BBs in alleviating ischemic symptoms and minimizing myocardial damage [6, 7]. Much of the earlier evidence supporting the clinical utility of BBs originated from an era preceding the widespread use of advanced diagnostic biomarkers and modern therapeutic interventions, such as percutaneous coronary intervention (PCI) [8–10]. Conversely, recent evidence, including findings from the Randomized Evaluation of Decreased Use of Beta‐Blockers after Acute Myocardial Infarction (REDUCE‐AMI) trial, has challenged the routine use of BBs in this patient subgroup, reporting no significant benefit over placebo in AMI patients with preserved left ventricular ejection fraction (LVEF) [11]. Similarly, the Clopidogrel and Metoprolol in Myocardial Infarction Trial (COMMIT), conducted between 1999 and 2005 and focused on early outcomes, did not support the use of BBs post‐MI [12]. The latest meta‐analysis of large RCTs using individual‐patient‐level data demonstrated that BB use did not reduce the incidence of all‐cause mortality, MI, or HF in patients withLVEF ≥ 50*%*following MI [13].

Given these controversies, clinicians face significant uncertainty in prescribing BBs for ACS patients, particularly those with LVEF ≥ 40*%*, after hospital discharge [14]. This study aims to provide a comprehensive, up‐to‐date systematic review and meta‐analysis of the effectiveness of long‐term BB use in ACS patients with different categories of non‐reduced LVEFs (≥ 40%, 40%–50%, and ≥ 50%), including both RCTs and observational studies.

## 2. Methods

This systematic review was conducted in accordance with the Preferred Reporting Items for Systematic Reviews and Meta‐Analyses (PRISMA) guidelines [15]. The review protocol was registered with the International Prospective Register of Systematic Reviews (PROSPERO) under the registration number CRD420251009171.

### 2.1. Search Resources and Strategy

A comprehensive literature search was conducted until September 2025 using four major electronic databases: PubMed, Scopus, Web of Science, and the Cochrane Central Register of Controlled Trials (CENTRAL). The search strategy was designed to capture studies investigating the use of BBs in patients with MI and their association with major adverse cardiovascular events (MACE). A combination of Medical Subject Headings (MeSH) and free‐text terms was used.

The BB component included the following terms: “adrenergic beta antagonist” OR “beta blocker ^∗^” OR “b‐blocker ^∗^” OR “beta antagonist ^∗^” OR “beta adrenoreceptor antagonist” OR “beta adrenergic receptor antagonist” OR “beta adrenergic blocking agent” OR “adrenergic beta‐1 receptor antagonists” OR acebutolol OR alprenolol OR atenolol OR betaxolol OR bisoprolol OR bunolol OR bupranolol OR bucindolol OR carteolol OR celiprolol OR carvedilol OR dihydroalprenolol OR esmolol OR iodocyanopindolol OR labetalol OR levobunolol OR metipranolol OR metoprolol OR nadolol OR nebivolol OR oxprenolol OR penbutolol OR practolol OR pindolol OR propranolol OR sotalol OR timolol.

The MI component included the following terms: “myocardial infarc ^∗^” OR “heart attack ^∗^” OR “cardiac infarc ^∗^” OR “cardiac arrest ^∗^” OR STEMI OR NSTEMI OR “myocardial ischemia” OR “cardiovascular stroke ^∗^” OR “cardiac stroke∗.”

The MACE component included the following terms: “major adverse cardiovascular event ^∗^” OR “cardiovascular event ^∗^” OR “cardiac event ^∗^” OR MACE OR “cardiovascular death ^∗^” OR “cardiac death ^∗^” OR “cardiac revascularization.”

Boolean operators (AND/OR) were used to combine the three search components appropriately. The complete and database‐specific search strings are provided in Table S1.

### 2.2. Eligibility Criteria

Eligible RCTs and observational studies were selected based on predefined PICO criteria: (1) Population: patients with ACS, including ST‐elevation myocardial infarction (STEMI), non‐ST‐elevation myocardial infarction (NSTEMI), or unstable angina, with baseline LVEF; (2) Intervention: treatment with one or more BBs alongside other standard therapies for ACS; (3) Comparison: treatment without BBs or placebo, in addition to standard ACS management; and (4) Outcomes: MACE, defined as cardiovascular‐related death, recurrent myocardial infarction (Re‐MI), stroke, and rehospitalization for HF. Additional outcomes included ACS, stable angina (chronic coronary syndrome [CCS]), and the need for revascularization. All‐cause mortality and angina severity were also considered.

### 2.3. Study Selection

All retrieved records were first imported into EndNote software, where duplicates were identified and removed by S.A.M. The deduplicated results were then uploaded into Rayyan, a web‐based platform designed for blinded screening in systematic reviews [16]. Three reviewers (S.A.S., A.S.H., and M.A.J.) independently and blindly screened the titles and abstracts of all studies. Full‐text articles of potentially eligible studies were subsequently retrieved and reviewed independently by the same three reviewers. A fourth reviewer (H.M.) assessed their work and resolved any discrepancies to ensure consistency and accuracy in the selection process.

### 2.4. Data Extraction

Data were independently extracted by five reviewers (S.A.S., P.A., M.A.J., A.S.H., and H.M.) into a structured Microsoft Excel spreadsheet and were cross‐checked by S.A.M. Data elements, predefined by S.A.M. and A.A., included study characteristics (country, study period, and inclusion and exclusion criteria), patient demographics (total population, mean age, sex distribution, and baseline LVEF), ACS definitions and diagnostic criteria, BB therapy details (type, initiation timing, and duration), other ACS interventions (PCI, coronary artery bypass grafting [CABG], and thrombolysis), and follow‐up data with clinical outcomes as defined in the eligibility criteria. Any discrepancies were resolved through discussion or final adjudication by S.A.M.

### 2.5. Risk of Bias Assessment

The risk of bias for each included study was independently assessed by four reviewers (S.A.S., H.M., A.S.H., and M.A.J.). For RCTs, the revised Cochrane Risk of Bias Tool (RoB 2.0) was used. This tool evaluates five domains of potential bias: (1) bias arising from the randomization process, (2) bias due to deviations from intended interventions, (3) bias due to missing outcome data, (4) bias in the measurement of the outcome, and (5) bias in the selection of the reported result. Each domain was judged as having low, high, or some concerns regarding bias, and an overall risk‐of‐bias judgment was assigned accordingly. For observational studies, the Risk Of Bias In Non‐randomized Studies of Interventions (ROBINS‐I) tool was applied. This tool assesses seven domains of bias: (1) bias due to confounding, (2) bias in selection of participants into the study, (3) bias in classification of interventions, (4) bias due to deviations from intended interventions, (5) bias due to missing data, (6) bias in measurement of outcomes, and (7) bias in selection of the reported result. Each domain was rated as low, moderate, serious, or critical risk of bias, or no information. Discrepancies between reviewers were resolved through discussion and consensus or by a final decision made by S.A.S. The detailed results and justifications for the risk‐of‐bias judgments are provided in the Supporting Information section.

### 2.6. Statistical Analysis

For RCTs, we used binary raw data consisting of the number of events and the total number of participants in both control and intervention arms to calculate pooled risk ratios (RRs) with corresponding 95% confidence intervals (CIs). For observational studies, we combined the precalculated hazard ratios (HRs) reported by the included studies.

Our predefined subgroup analysis was based on LVEF categories: ≥ 50%, 40%–49%, and > 40%. Subgroup analyses were performed for each outcome reported by at least four studies. Heterogeneity was assessed using the chi‐square (*χ* ^2^) test and Higgins’ *I* ^2^ statistic, with *I* ^2^ values exceeding 50% considered to be substantial heterogeneity.

We did not generate funnel plots to assess publication bias because the number of studies for each outcome was fewer than 10, which reduces the statistical power of funnel plot asymmetry tests. All analyses were performed using R Version 4.4.3, and*p* values < 0.05were considered statistically significant.

## 3. Results

**Figure 1:**
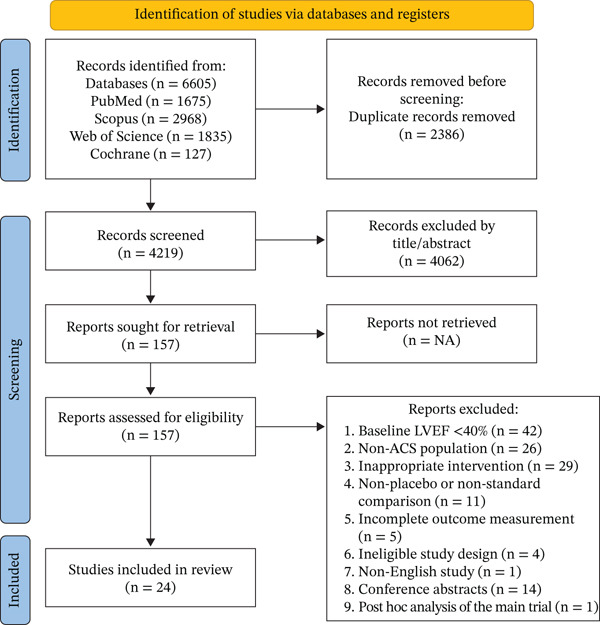
PRISMA flowchart of the study.

A total of 6605 studies were identified through searches in PubMed (*n* = 1675), Scopus (*n* = 2968), Web of Science (*n* = 1835), and Cochrane CENTRAL (*n* = 127). After removing 2386 duplicates, 4219 records remained for title and abstract screening, from which 157 studies were selected for full‐text review. Of these articles, 133 studies were excluded for the following reasons: baseline LVEF of less than 40% (*n* = 42), non‐ACS population (*n* = 26), inappropriate intervention (*n* = 29), non‐placebo or non‐standard comparison (*n* = 11), incomplete outcome measurement (*n* = 5), ineligible study design (*n* = 4), non‐English study (*n* = 1), conference abstracts (*n* = 14), and post hoc analysis of the main trial (*n* = 1). Ultimately, 24 studies were included in the final analysis. The study selection process is illustrated in the PRISMA flow diagram (Figure 1).

### 3.1. Baseline Characteristics

Of the 24 included studies, 19 were observational, and five were RCTs. Five studies specifically enrolled patients with LVEF ≥ 50*%*, 12 included those with LVEF > 40*%*, and seven involved patients with various LVEF classifications. Among the RCTs, one focused on patients with LVEF ≥ 50*%* [8], three focused on patients with LVEF > 40*%* [1, 17, 18], and one included participants across multiple LVEF categories [19]. Among the observational studies, four focused on patients with LVEF ≥ 50*%* [20–23], nine focused on patients with LVEF > 40*%* [24–32], and six included participants across multiple LVEF categories [33–38]. In total, 118,612 patients were included, comprising 85,413 (72.0%) BB users and 33,199 (28.0%) non‐BB users across 24 studies. A summary of baseline characteristics at both the study and overall levels is provided in Tables 1 and 2.

**Table 1:** Baseline characteristics of included studies by LVEF category.

LVEF category	First author (year)	Study design	*N*(BB)	*N*(non‐BB)	Age, median (BB)	Age, median (non‐BB)	Male % (BB)	Male % (non‐BB)	STEMI % (BB)	STEMI % (non‐BB)	NSTEMI % (BB)	NSTEMI % (non‐BB)	PCI % (BB)	PCI % (non‐BB)	CABG % (BB)	CABG % (non‐BB)	Follow‐up, median (years)	Most common BB
≥ 50%	Yndigegn (2024) REDUCE‐AMI [8]	RCT	2508	2512	65	65	77.6	77.4	35.2	35.5	64.8	64.5	95.8	95.2	3.5	4.1	3.5	Metoprolol
	Choo (2014) [21]	Observational	2424	595	60.9	63.1	74.2	69.2	85.3	57.5	14.7	42.5	100	NA	NA	NA	3	Carvedilol
	Chen (2021) [20]	Observational	2067	337	57.3	60.4	81	82.2	84.6	90.8	15.4	9.2	100	NA	NA	NA	1.99	Metoprolol
	Wen (2022) [23]	Observational	2049	470	61.7	61.7	79.3	80.4	64.6	63.2	35.4	36.8	81.6	70.4	0.1	0.4	3.61	Metoprolol
	Nishihira (2024) [22]	Observational	192	288	84.1	NA	76	NA	91.7	NA	8.3	NA	NA	NA	NA	NA	3.12	NA

> 40%	Silvain (2024) ABYSS [1]	RCT	1852	1846	63.5	63.5	82.7	82.9	62.7	63.3	37.3	36.7	96.4	97.4	4.7	3.5	3	Bisoprolol
	Munkhaugen (2025) BETAMI‐DANBLOCK [18]	RCT	2783	2791	63.9	63.5	78.4	79.9	47.8	47.2	52.2	52.8	92.9	92.5	1.7	2	3.7	Metoprolol
	Ibanez (2025) REBOOT [17]	RCT	4207	4231	61.4	61.3	80.6	80.8	51	50.8	49	49.2	93.5	93.7	0.1	0.2	3.7	Bisoprolol
	Ozasa (2013) [25]	Observational	1117	4171	68.2	68	71	72	NA	NA	NA	NA	100	NA	NA	NA	3	Carvedilol
	Puymirat (2016) [26]	Observational	2050	629	63.7	68	74	67	57	53	43	47	39	35	3	4	5	Atenolol
	Konishi (2016) [29]	Observational	197	227	64	64.2	77.2	81.1	100	NA	0	NA	100	NA	NA	NA	4.7	NA
	Abi Khalil (2017) [30]	Observational	3520	668	55	56	81.7	75.1	45.5	48	54.5	52	20.7	9.7	9.2	NA	1	NA
	Chen (2020) [27]	Observational	5043	588	57.8	60.3	76.2	80	NA	NA	NA	NA	100	NA	NA	NA	2	NA
	El Nasasra (2021) [28]	Observational	6007	1385	60.8	62.2	79.2	78.3	47.2	NA	52.8	NA	24.2	17.4	4.6	1	NA	NA
	Joo (2022) [24]	Observational	2508	396	63.8	67.4	74.1	68.4	62.7	51.3	37.3	48.7	NA	NA	NA	NA	2	Carvedilol
	Fukase (2022) [31]	Observational	514	504	72	72	75	78	NA	NA	NA	NA	100	NA	NA	NA	5.1	Bisoprolol
	Johner (2024) [32]	Observational	1758	319	62.6	62.8	79.4	79.3	55.2	49.2	44.8	50.8	93.8	NA	0.3	NA	NA	NA

Various/mixed	Watanabe (2018) CAPITAL‐RCT [19]	RCT	394	400	63.9	64.5	83	78	100	NA	0	NA	100	100	0	0	3.7	Carvedilol
	Bao (2012) [33]	Observational	1614	2078	65.8	68	77.8	72.2	100	NA	0	NA	100	NA	NA	NA	3	Carvedilol
	Yang (2014) [38]	Observational	6873	1637	62.7	65.6	75.4	74.3	100	NA	0	NA	100	NA	NA	NA	1	NA
	Lee (2015) [36]	Observational	598	303	56	61	82.1	74.3	100	NA	0	NA	100	NA	NA	NA	4.5	Carvedilol
	Joo (2020) [35]	Observational	10,251	1949	63.2	65.6	74.7	72.4	47.6	NA	52.4	NA	NA	NA	NA	NA	1	Bisoprolol
	Peck (2021) [37]	Observational	14,636	2926	63.2	65.4	76.9	73.4	47.4	33.8	52.6	66.2	100	NA	NA	NA	5.3	NA
	Boo (2024) [34]	Observational	10,251	1949	63.2	65.6	74.7	72.4	49.4	38	50.6	62	93	75.6	1.2	2.2	3	Bisoprolol

*Note:* Data are presented as the number of patients in the beta‐blocker (BB) and non‐BB groups, along with baseline characteristics including age, sex, proportions of STEMI/NSTEMI, rates of PCI and CABG, follow‐up duration, and the most commonly prescribed beta‐blocker. Continuous variables are reported as mean or median values as provided in the original studies, and categorical variables are expressed as percentages.

Abbreviations: BB, beta‐blocker; CABG, coronary artery bypass grafting; LVEF, left ventricular ejection fraction; *N*, number; NA, not available; non‐BB, no beta‐blocker; NSTEMI, non‐ST‐elevation myocardial infarction; PCI, percutaneous coronary intervention; RCT, randomized controlled trial; STEMI, ST‐elevation myocardial infarction.

**Table 2:** Summary of baseline characteristics by study design.

Study design	*N*(BB)	*N*(non‐BB)	Age (BB), median (IQR)	Age (non‐BB), median (IQR)	Male % (BB), median (IQR)	Male % (non‐BB), median (IQR)	STEMI % (BB), median (IQR)	STEMI % (non‐BB), median (IQR)	NSTEMI % (BB), median (IQR)	NSTEMI % (non‐BB), median (IQR)	PCI % (BB), median (IQR)	PCI % (non‐BB), median (IQR)	CABG % (BB), median (IQR)	CABG % (non‐BB), median (IQR)	Follow‐up (years), median (IQR)	Most common BB
RCT	11,744	11,780	63.9 (63.5–63.9)	63.5 (63.5–64.5)	80.6 (78.4–82.7)	79.9 (78–80.8)	51 (47.8–62.7)	49 (44.28–53.92)	49 (37.3–52.2)	51 (46.08–55.72)	95.8 (93.5–96.4)	95.2 (93.7–97.4)	1.7 (0.1–3.5)	2 (0.2–3.5)	3.7 (3.5–3.7)	Bisoprolol 2 (40%), metoprolol 2 (40%); NR 1 (20%)
Observational	73,669	21,419	63.2 (60.85–63.9)	64.8 (61.83–66.95)	76.2 (74.7–79.25)	74.3 (72.25–79.05)	63.65 (48.95–93.78)	51.3 (48–57.5)	36.35 (6.23–51.05)	48.7 (42.5–52)	100 (90.15–100)	35 (17.4–70.4)	2.1 (0.52–4.2)	1.6 (0.85–2.65)	3 (2–4.5)	Carvedilol 5 (28%), metoprolol 2 (11%); NR 7 (39%)
All	85,413	33,199	63.2 (61.27–63.92)	64.2 (61.95–65.6)	77.4 (74.92–79.7)	77.4 (72.4–79.95)	62.7 (47.8–91.7)	50.8 (47.2–57.5)	37.3 (8.3–52.2)	49.2 (42.5–52.8)	100 (93–100)	84.05 (43.85–94.83)	1.7 (0.2–4.05)	2 (0.4–3.5)	3.06 (2.25–3.7)	Carvedilol 6 (26%), metoprolol 4 (17%); NR 8 (35%)

*Note:* The data represent aggregated summaries across included studies. Continuous variables are reported as median with interquartile range (IQR) across studies, and categorical variables as median percentage (IQR).

Abbreviations: BB, beta‐blocker; CABG, coronary artery bypass grafting; non‐BB, no beta‐blocker; NSTEMI, non‐ST‐elevation myocardial infarction; PCI, percutaneous coronary intervention; RCT, randomized controlled trial; STEMI, ST‐elevation myocardial infarction.

### 3.2. RCTs’ Baseline Characteristics

Among the five RCTs, Yndigegn (2024) REDUCE‐AMI [8], Silvain (2024) ABYSS [1], Munkhaugen (2025) BETAMI‐DANBLOCK [18], Ibanez (2025) REBOOT [17], and Watanabe (2018) CAPITAL‐RCT (Carvedilol Post‐Intervention Long‐Term Administration in a Large‐Scale Randomized Controlled Trial) [19], a total of 23,524 patients were analyzed, including 11,744 BB users and 11,780 non‐BB users. The mean age of BB users in RCTs ranged from 61.4 to 65 years, and that of non‐BB users from 61.3 to 65.4 years. The proportion of male participants varied between 77.6% and 83% among BB users and between 77.4% and 82.9% among non‐BB users. The prevalence of STEMI ranged from 47.8% to 100%, with PCI performed in 92.9%–100% and CABG in 0%–4.7% of participants. The most commonly prescribed BBs were metoprolol, bisoprolol, and carvedilol. Follow‐up durations in RCTs ranged from 3 to 3.7 years (Tables 1 and 2).

### 3.3. Observational Studies’ Baseline Characteristics

In the 19 observational studies, a total of 95,088 patients were included, comprising 73,669 BB users and 21,419 non‐BB users. The mean age of BB users ranged from 55 to 84 years, and that of non‐BB users from 56 to 68 years. The proportion of male participants varied between 71% and 83% among BB users and between 67% and 82% among non‐BB users. The frequency of STEMI ranged from 33.8% to 100%, while PCI was performed in 24.2%–100% and CABG in 0.1%–9.2% of patients. The predominant BBs were metoprolol, bisoprolol, and carvedilol. The follow‐up duration in observational studies ranged from 1 to 5.3 years (Tables 1 and 2).

### 3.4. Risk of Bias

**Figure 2:**
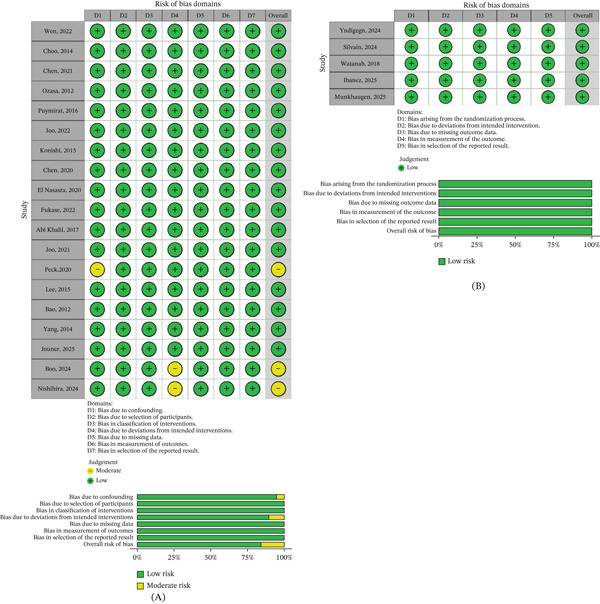
Quality assessment of included (A) observational studies and (B) RCTs.

Of the 19 observational studies, three were classified as having a moderate risk of bias: one due to confounding (Domain 1) [37] and two due to the selection of participants (Domain 4) [22, 34], while the remaining studies were deemed to have a low risk of bias (Figure 2A). Additionally, all RCTs were assessed as having a low risk of bias (Figure 2B).

### 3.5. All‐Cause Mortality

**Figure 3:**
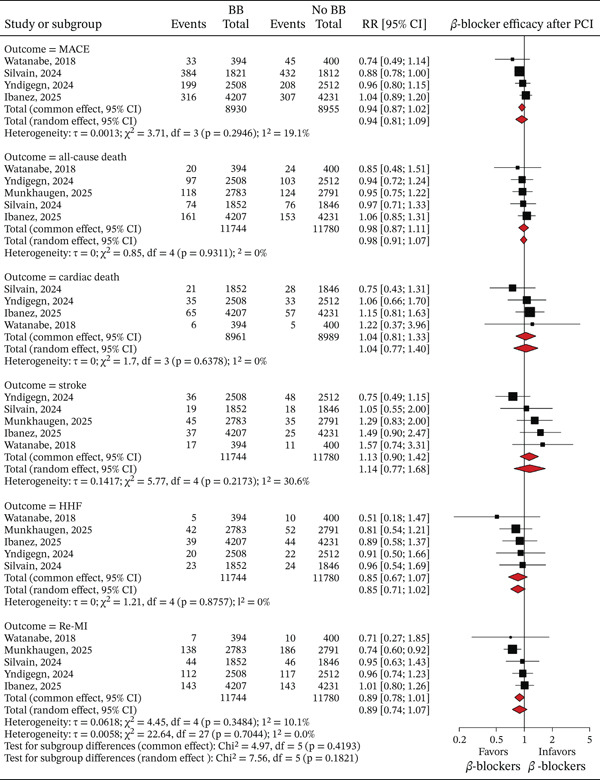
Forest plot of all endpoint outcomes across RCTs: Yndigegn (2024) REDUCE‐AMI [8], Silvain (2024) ABYSS [1], Munkhaugen (2025) BETAMI‐DANBLOCK [18], Ibanez (2025) REBOOT [17], and Watanabe (2018) CAPITAL‐RCT [19].

**Figure 4:**
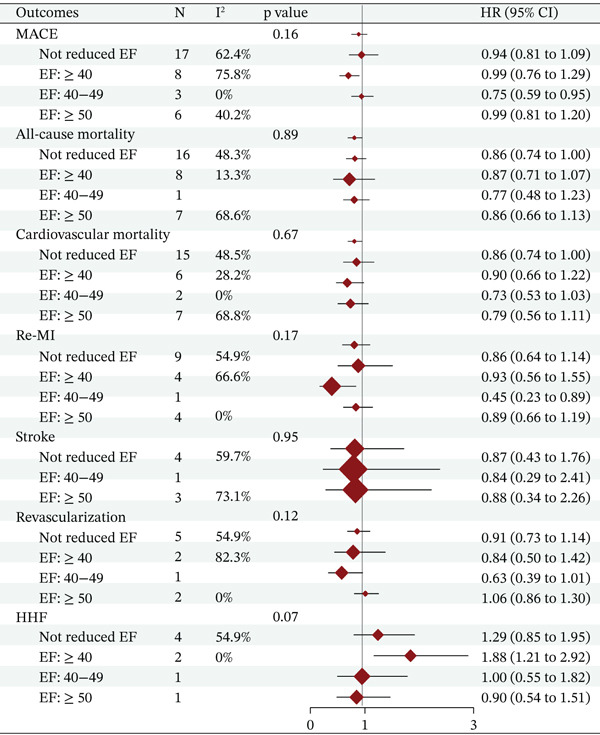
Pooled hazard ratios (HRs) with 95% confidence intervals (CIs) for observational studies, stratified by left ventricular ejection fraction (LVEF). *N* indicates the number of included studies, *I* ^2^ represents heterogeneity, and the *p* value refers to the test for subgroup differences.

A meta‐analysis of five RCTs showed non‐significant reductions in all‐cause mortality between BB and non‐BB arms (RR = 0.98, 95*%*CI = 0.87–1.11, *I* ^2^ = 0*%*) (Figure 3). Aligning with the RCTs, aggregated observational study data suggested a decreasing trend in all‐cause mortality for BB users (HR = 0.86, 95*%*CI = 0.74–1.00, *I* ^2^ = 46.3*%*). Furthermore, LVEF‐stratified subgroup analyses yielded non‐significant findings among BB users with LVEF ≥ 50*%* (HR = 0.86, 95*%*CI = 0.66–1.13, *I* ^2^ = 68.6*%*) and LVEF > 40*%* (HR = 0.87, 95*%*CI = 0.71–1.07, *I* ^2^ = 13.3*%*) (Figure 4, Figure S1).

### 3.6. MACE

The pooled analysis of four RCTs indicated a non‐significant reduction in MACE (RR = 0.94, 95*%*CI = 0.87–1.02, *I* ^2^ = 19.1*%*) (Figure 3). Similarly, the pooled results from the observational studies implied a non‐significant minimal reduction in MACE incidence (HR = 0.94, 95*%*CI = 0.81–1.09, *I* ^2^ = 62.4*%*). Further subgroup analyses stratified by LVEF revealed a statistically significant reduction in MACE among patients with LVEF 40%–49% (HR = 0.75, 95*%*CI = 0.59–0.95, *I* ^2^ = 0*%*). However, no significant reductions were observed in those with LVEF > 40*%* (HR = 0.99, 95*%*CI = 0.76–1.29, *I* ^2^ = 75.8*%*) or LVEF ≥ 50*%* (HR = 0.99, 95*%*CI = 0.81–1.20, *I* ^2^ = 40.2*%*), and the test for subgroup differences was non‐significant (*p* = 0.165) (Figure 4, Figure S2).

### 3.7. Cardiovascular Mortality

Four RCTs demonstrated comparable cardiovascular mortality rates between BB and non‐BB arms (RR = 1.04, 95*%*CI = 0.77–1.40, *I* ^2^ = 0*%*) (Figure 3). Pooled observational data indicated a non‐significant reduction (HR = 0.82, 95*%*CI = 0.67–1.00, *I* ^2^ = 48.5*%*). Across LVEF subgroups, outcomes remained neutral: LVEF ≥ 50*%* (HR = 0.79, 95*%*CI = 0.56–1.11, *I* ^2^ = 68.8*%*), LVEF > 40*%* (HR = 0.90, 95*%*CI = 0.66–1.22, *I* ^2^ = 28.2*%*), and LVEF 40%–49% (HR = 0.73, 95*%*CI = 0.53–1.03, *I* ^2^ = 0*%*), supported by a non‐significant subgroup difference (*p* = 0.66) (Figure 4, Figure S3).

### 3.8. Stroke

Analysis across five RCTs found stroke rates unaffected by long‐term BB use (RR = 1.14, 95*%*CI = 0.77–1.68, *I* ^2^ = 30.6*%*) (Figure 3). Similarly, three observational studies mirrored this, with a non‐significant risk reduction (HR = 0.87, 95*%*CI = 0.43–1.76, *I* ^2^ = 59.7*%*). In subgroup analysis by LVEF, BB use in patients with LVEF ≥ 50*%* also showed no significant association (HR = 0.88, 95*%*CI = 0.34–2.26, *I* ^2^ = 73.1*%*) (Figure 4, Figure S4).

### 3.9. Hospitalization for Heart Failure (HHF)

The risk of HHF did not differ significantly between BB and non‐BB groups in five RCTs (RR = 0.85, 95*%*CI = 0.67–1.07, *I* ^2^ = 0*%*) (Figure 3). Consistent findings were observed across four observational studies, showing no significant association between long‐term BB use and HHF (HR = 1.29, 95*%*CI = 0.85–1.95, *I* ^2^ = 45*%*) (Figure 4, Figure S5).

### 3.10. Re‐MI

Long‐term BB use in five RCTs yielded no substantial Re‐MI risk reduction (RR = 0.89, 95*%*CI = 0.78–1.01, *I* ^2^ = 10.1*%*) (Figure 3). Pooled results from eight observational studies corroborated this finding, yielding a non‐significant association (HR = 0.86, 95*%*CI = 0.64–1.14, *I* ^2^ = 54.9*%*). LVEF subgroups were similarly inconclusive: LVEF ≥ 50*%* (HR = 0.89, 95*%*CI = 0.66–1.19, *I* ^2^ = 0*%*) or LVEF > 40*%* (HR = 0.93, 95*%*CI = 0.56–1.55, *I* ^2^ = 66.6*%*). Only one study evaluated the effect of BB use on Re‐MI in patients with LVEF between 40% and 50% (HR = 0.45, 95*%*CI = 0.23–0.89) (Figure 4, Figure S6).

### 3.11. Revascularization

Analyzing four observational studies, revascularization risks appeared similar between long‐term BB users and non‐BB users (HR = 0.91, 95*%*CI = 0.73–1.14, *I* ^2^ = 59.6*%*) (Figure 4, Figure S7).

### 3.12. Sensitivity Analyses

Sensitivity analyses excluding studies with a moderate risk of bias demonstrated consistent findings with the primary analyses. The observed reductions in MACE and Re‐MI among patients with LVEF 40%–49% remained unchanged. Estimates for all other outcomes were largely similar to the main analysis, except for all‐cause mortality in the overall non‐reduced EF group, where the effect estimate became more pronounced and reached statistical significance (HR = 0.81, 95*%*CI = 0.67–0.98 vs. HR = 0.86, 95*%*CI = 0.74–1.00). Overall, these results reinforce the stability of the findings (Table S2).

### 3.13. Certainty of Evidence

The certainty of evidence, assessed using the GRADE approach, was rated as moderate for all primary and secondary outcomes derived from RCTs, primarily due to imprecision with CIs crossing the null. In contrast, the certainty of evidence for the LVEF 40%–49% subgroup was rated as low, reflecting reliance on observational data, potential residual confounding, and indirectness due to the lack of dedicated RCT subgroup analyses. Detailed GRADE assessments are provided in Table S3.

## 4. Discussion

This meta‐analysis evaluated the therapeutic efficacy of post‐ACS BB administration in individuals with preserved LVEFs classified as ≥ 50%, between 40% and 49%, and > 40% by analyzing the results of RCTs and observational studies. Our analyses indicated an overall neutral effect of long‐term BB use on all‐cause mortality, cardiac mortality, revascularization, MACE (cardiovascular‐related death, Re‐MI, stroke, and rehospitalization for HF), and its components in this subgroup of patients. Notably, our findings indicated that patients with LVEFs between 40% and 49% may derive potential benefits from BB use regarding MACE, as per the pooled analysis of the observational studies. Moreover, in this understudied subgroup (LVEF 40%–49%), an RCT showed a preventive effect for Re‐MI. These findings demonstrate a neutral effect of long‐term use of BBs in patients with preserved LVEF, especially patients with LVEF > 50*%*, and highlight the need for focused investigation of patients with LVEF between 40% and 50%.

The current guidelines report on the scant evidence supporting the extended use of BBs post‐MI in patients with preserved EF, acknowledging uncertainty concerning the potential benefits of BB use in patients with preserved EFs [5]. Mirroring our findings, multiple meta‐analyses have indicated no additional benefit in preventing all‐cause mortality, cardiovascular death, or MACE following routine use of BBs in patients with LVEF ≥ 50*%* following MI [39, 40]. Similarly, although the ACC/AHA guideline recommends BB use for individuals with reduced EF, it advises against long‐term BB administration in cases of preserved EFs without other indications [3, 4]. Comparable to our findings of potential benefits in patients with EFs between 40% and 49%, the ESC and ACC/AHA highlight their cautious stance on BB administration in individuals without HF and with mildly reduced EFs, highlighting a possible benefit in this population [3–5]. Moreover, a recent individual patient data meta‐analysis of RCTs confirms this benefit of BB use in preventing the composite of all‐cause death, new MI, or HF in this population [41].

The CAPITAL‐RCT, which was a prospective, multicenter, open‐label RCT in Japan, found no significant advantages of long‐term BB therapy in patients with LVEF ≥ 40*%* regarding all‐cause mortality and MI post‐PCI, corroborating the findings of the present meta‐analysis [42]. Similarly, the REDUCE‐AMI, a multicenter, open‐label, registry‐based RCT, further supported our findings, as it observed non‐significant differences between BB and non‐BB users concerning all‐cause mortality and cardiovascular death and concluded that there were no overall benefits in patients with preserved EF who were previously treated with contemporary revascularization [8]. Interestingly, the ABYSS trial investigated the primary composite outcomes (all‐cause mortality, Re‐MI, stroke, and cardiovascular hospitalization) between the BB continuation and discontinuation arms and exhibited increased primary outcomes in the discontinuation group, prominently driven by the cardiovascular hospitalization outcome, with similar non‐significant results on death, MI, or stroke in patients with LVEF ≥ 40*%* [43].

In addition to large trials, two extensive nationwide cohort studies reported no significant differences between BB users and controls concerning all‐cause mortality, MI, and revascularization among MI patients without HF, which is in accordance with the findings of this study [44, 45]. Moreover, contrasting with the reduced risk of MACE pooled from RCTs, Asano et al. [46] evaluated the efficacy of long‐term BB use in individuals with 40*%* < EF < 49*%* post‐PCI and reported no significant differences between BB and non‐BB recipients concerning cumulative all‐cause mortality and non‐fatal ACS, with inconsistencies arising due to variations in the non‐fatal ACS and MACE definitions. Taken together, our results largely align with prior research, indicating a nuanced benefit–risk profile of long‐term BB use in patients with preserved EFs concerning all‐cause and cardiovascular mortality, Re‐MI, and MACE.

Interestingly, one meta‐analysis on post‐MI patients without HF and with LVEF ≥ 40*%* observed a temporal diminishing effect of BBs on mortality rates, more pronounced since 2010, potentially attributed to the advancements in PCI and revascularization techniques, and reported increased cardiovascular mortality and MACE incidence associated with long‐term BB use, which contrasts with our findings of a neutral and decreasing effect on survival outcomes and MACE in patients with preserved EF, respectively, possibly due to the confounding‐by‐indication bias, as BBs are commonly prescribed for patients with various comorbidities [46, 47]. Additionally, in contrast to Chi et al.’s [47] study, the current meta‐analysis employed a stepwise stratification of LVEF to enhance the estimation of associations. A recent meta‐analysis of RCTs evaluating the efficacy of BBs in post‐MI patients without HF and with LVEF > 40*%* demonstrated potential benefits of BB use in reducing all‐cause mortality. This contrasts with our findings, which may be attributed to differences in study design. While the aforementioned review exclusively included RCTs and excluded patients with HF, it included participants classified as having Class I/II HF [48]. Furthermore, another meta‐analysis evaluating the long‐term effects of BB use in patients with preserved EFs reported no overall benefits regarding all‐cause mortality, MACE (non‐fatal MI, cardiovascular death, and hospitalization for unstable angina or HF), and cardiovascular mortality, further reinforcing our findings [39].

Beta‐receptors primarily exist as beta‐1 (B1), beta‐2 (B2), and beta‐3 (B3) receptors, with B1s commonly known as mediators of cardiac activity and B2s exhibiting a wider range of activities, including metabolic and smooth muscle effects [49, 50]. Conversely, B3s are more active in the breakdown of fat cells [49, 50]. Common activators of B1, including catecholamines such as epinephrine and norepinephrine, increase cardiac automaticity, conduction velocity, and renin release, thereby increasing blood pressure [51, 52]. Once BBs bind to B1, they induce their inhibitory, chronotropic, and inotropic effects, slow heart rate, and result in reduced cardiac output, myocardial oxygen demand, and blood pressure via reduced renin, justifying their administration in patients with reduced EFs, in contrast to individuals with preserved EFs, for whom the reduced heart rate and cardiac output may even be detrimental, especially during high oxygen–demanding activities [51, 52].

In post‐ACS patients with LVEF 40%–49% (HFmrEF), residual sympathetic hyperactivity and subclinical adverse remodeling persist even without overt HF, with elevated catecholamine drive promoting myocyte apoptosis, interstitial fibrosis, increased wall stress, and heightened vulnerability to recurrent ischemia and arrhythmias [53, 54]. Beta‐blockers counteract this neurohormonal overactivation by blunting B1‐mediated chronotropic and inotropic effects, thereby reducing myocardial oxygen demand and slowing progression toward further EF decline—providing a mechanistic basis for the observed reductions in MACE and Re‐MI in this subgroup [55]. In contrast, once LVEF reaches ≥ 50% (HFpEF), neurohormonal activation is minimal, compensatory mechanisms remain intact, and the Frank–Starling relationship functions efficiently without pathologic remodeling pressure; in this setting, the same negative chronotropic and inotropic actions of BBs can unnecessarily lower cardiac output, impair exercise tolerance, and confer no incremental benefit, thereby explaining the consistent neutral effect on mortality, MACE, and its components [56–58]. Furthermore, in patients with preserved EF (≥ 50%), these negative chronotropic and inotropic effects may manifest as symptomatic bradycardia, fatigue, or reduced exercise tolerance, potentially outweighing any marginal benefit and contributing to lower treatment adherence [59].

This meta‐analysis is the first to incorporate a substantial number of RCTs and observational cohort studies, focusing solely on patients with non‐reduced EFs, in contrast to previous meta‐analyses that featured a smaller number of studies [39, 45, 47, 60]. Additionally, the present study provides a guideline‐based heterogeneity analysis, previously outlined in the European and American guidelines [4, 5]. Moreover, based on available evidence, this is the first meta‐analysis to examine the association between BB use and clinical endpoint outcomes stratified by EF levels, suggesting a possible benefit in patients with EF levels of 40%–49%, while the effect appears neutral in those with EF ≥ 50*%*. This meta‐analysis has some limitations. The broad MACE definition, which included softer endpoints such as revascularization and HHF, may have diluted potential effects on harder outcomes (cardiovascular death and Re‐MI). However, all individual outcomes—both hard and soft—were also evaluated separately, allowing for a more granular assessment of treatment effects across endpoint types. Heterogeneity was generally low to moderate in the RCT analyses (*I* ^2^ ≤ 30.6*%*), but some observational outcomes showed moderate to substantial heterogeneity (e.g., *I* ^2^ = 62.4*%* for MACE), likely due to differences in BB type and dosage, variations in the definitions of endpoint outcomes, treatment and follow‐up durations (from 1 to 5.3 years), and revascularization rates across studies. Sensitivity analyses excluding the three observational studies with moderate risk of bias yielded virtually unchanged pooled estimates for all key outcomes, supporting the robustness of our findings. Furthermore, 85.7% of the included studies were observational, rendering them prone to immortal time bias, residual confounding despite adjustments, and potential overestimation of treatment effects. Differences in background therapy may also limit generalizability to contemporary populations. Finally, the lack of patient‐level data prevented exploration of dose–response relationships or detailed subgroup interactions.

Collectively, this meta‐analysis further reinforces the notion that long‐term BB use in patients with preserved EFs and without HF may provide no overall benefits. However, there may be possible advantages in patients with EFs between 40% and 49%. Nevertheless, this potential benefit should be interpreted with caution, as the significant MACE reduction derives exclusively from observational studies prone to residual confounding and selection bias, while the Re‐MI benefit is based on a single study, rendering the findings promising yet underpowered and at risk of overinterpretation. Clinicians may reasonably continue long‐term BBs in post‐ACS patients with LVEF 40%–49% in the absence of contraindications, given the suggestive observational data and potential mechanistic benefit; however, decisions should remain individualized, weighing possible harms such as bradycardia and fatigue against the unproven long‐term benefit pending dedicated randomized trials. This emphasizes the need for individualized clinical decisions and further studies to clarify these findings.

## 5. Conclusion

Post‐ACS long‐term BB administration did not demonstrate significant benefits regarding mortality, revascularization, MACE, or stroke in patients with preserved EF (LVEF ≥ 50*%*). In the LVEF 40%–49% subgroup, observational data suggested a possible reduction in MACE and Re‐MI; however, these findings were derived primarily from observational studies and therefore require confirmation in dedicated RCTs. Clinicians should weigh potential benefits against possible harms such as bradycardia, fatigue, and reduced exercise tolerance when considering BB use in this population. Larger, well‐powered RCTs are needed to clarify the role of BBs across the spectrum of non‐reduced EF.

**Nomenclature**
BB: beta‐blocker
AMI: acute myocardial infarction
ACS: acute coronary syndrome
RCT: randomized controlled trial
PCI: percutaneous coronary intervention
LVEF: left ventricular ejection fraction
EF: ejection fraction
MACE: major adverse cardiovascular events
STEMI: ST‐elevation myocardial infarction
NSTEMI: non‐ST‐elevation myocardial infarction
HF: heart failure
HHF: hospitalization for heart failure
HFmrEF: heart failure with mildly reduced ejection fraction (LVEF 41–49%)
HFpEF: heart failure with preserved ejection fraction (LVEF ≥ 50*%*)
CABG: coronary artery bypass grafting
CCS: chronic coronary syndrome

## Author Contributions

A.A. and S.A.M. contributed to conceptualization and design. The search strategy was developed by R.M. and S.A.M. Data collection was performed by S.A.S., P.A., A.S.H., H.M., and M.A.J. Data analysis was performed by D.S.N. and P.A. The primary draft was written by S.A.M., S.A.S., H.M., E.R., D.S.N., and M.R. Revisions were performed by S.A.M., P.A., E.R., D.S.N., W.S.A., R.G., and A.A.

## Funding

No funding was received for this manuscript.

## Disclosure

All the listed authors have contributed substantially to the manuscript and have agreed to the final submitted version.

## Ethics Statement

The authors have nothing to report.

## Conflicts of Interest

The authors declare no conflicts of interest.

## Supporting information


**Supporting Information** Additional supporting information can be found online in the Supporting Information section. Table S1: Search strategy and search string details. Table S2: Sensitivity analysis. Table S3: Certainty of evidence. Figures S1, S2, S3, S4, S5, S6, and S7: Meta‐analysis results of observational studies.

## Data Availability

All data analyzed during the study are included in this manuscript and its supporting information.
