# Supplementary material for: Baseline Ejection Fraction as a Modifier of Beta‐Blocker Therapeutic Effects in Post‐Acute Coronary Syndrome Patients With Non‐Reduced Ejection Fraction: A Systematic Review and Meta‐Analysis
Source: Cardiovasc Ther. 2026 Jun 17;2026:2988999. doi: 10.1155/cdr/2988999 (PMC13276276; doi:10.1155/cdr/2988999)

**Title**

**Baseline Ejection Fraction as a Modifier of Beta-Blocker Therapeutic Effects in Post-Acute Coronary Syndrome Patients with Non-Reduced Ejection Fraction: A Systematic Review and Meta-Analysis**

**Short title:** B-Blockers in ACS with Non-Reduced EF

**Authors**

Seyed Alireza Mirhosseini^1,2^, Seyed Amir Sadrzadeh^3^, Davood Semirani-Nezhad^4^, Erta Rajabi^5^, Hanieh Mohammadi^3^, Alireza Shaabanpoor Haghighi^3^, Mohammad Amin Jamali^3^, Reza Moshfeghinia^3^, Maryam Ranjbar^6^, Pouria Azami^1,2^, Rahul Gupta^7^, Wilbert S. Aronow^8^, Armin Attar^2^

^1^ Cardiovascular Research Center, Shiraz University of Medical Sciences, Shiraz, Iran

^2^ Department of Cardiovascular Medicine, TAHA Clinical Trial Group, School of Medicine, Shiraz University of Medical Sciences, Shiraz, Iran

^3^ Student Research Committee, Shiraz University of Medical Sciences, Shiraz, Iran

^4^ Tehran Heart Center, Cardiovascular Diseases Research Institute, Tehran University of Medical Sciences, Tehran, Iran

^5^ Faculty of Medicine, Tehran University of Medical Sciences, Tehran, Iran

^6^ MD-MPH Department, School of Medicine, Shiraz University of Medical Sciences, Shiraz, Iran

^7^ Department of Cardiology, St. Luke’s University Health Network, Bethlehem, PA, USA

^8^ Department of Medicine, Westchester Medical Center and New York Medical College

***Corresponding author:** Armin Attar, MD, PhD.

**Address**: Department of Cardiovascular Medicine, TAHA clinical trial group, School of Medicine, Zand Street, Shiraz University of Medical Sciences, Postal Code: 71344-1864, Shiraz, Iran.

**Email**: [attar_armin@yahoo.com](mailto:attar_armin@yahoo.com), **Phone No**: +989177141797, **Fax**: +987112349521

**Supplementary Document**

# **Supplementary Table 1. Search Strategy**

| Database | Search strategy | N |
| --- | --- | --- |
| PubMed | ((((((((((((((((((((((((((((((((((("adrenergic beta antagonist"[Title/Abstract]) OR ("beta blocker*"[Title/Abstract])) OR ("b-blocker*"[Title/Abstract])) OR ("beta antagonist*"[Title/Abstract])) OR ("Adrenergic beta-Antagonists"[Mesh])) OR ("beta adrenoreceptor antagonist"[Title/Abstract])) OR ("beta adrenergic receptor antagonist"[Title/Abstract])) OR ("beta adrenergic blocking agent"[Title/Abstract])) OR ("adrenergic beta-1 receptor antagonists"[Title/Abstract])) OR (acebutolol[Title/Abstract])) OR (alprenolol[Title/Abstract])) OR (atenolol[Title/Abstract])) OR (betaxolol[Title/Abstract])) OR (bisoprolol[Title/Abstract])) OR (bunolol[Title/Abstract])) OR (bupranolol[Title/Abstract])) OR (Bucindolol[Title/Abstract])) OR (carteolol[Title/Abstract])) OR (celiprolol[Title/Abstract])) OR (Carvedilol[Title/Abstract])) OR (dihydroalprenolol[Title/Abstract])) OR (esmolol[Title/Abstract])) OR (iodocyanopindolol[Title/Abstract])) OR (labetalol[Title/Abstract])) OR (levobunolol[Title/Abstract])) OR (metipranolol[Title/Abstract])) OR (metoprolol[Title/Abstract])) OR (nadolol[Title/Abstract])) OR (Nebivolol[Title/Abstract])) OR (oxprenolol[Title/Abstract])) OR (penbutolol[Title/Abstract])) OR (practolol[Title/Abstract])) OR (pindolol[Title/Abstract])) OR (propranolol[Title/Abstract])) OR (sotalol[Title/Abstract])) OR (timolol[Title/Abstract])  ((((((((("Myocardial Infarction"[Mesh]) OR ("Myocardial infarc*"[Title/Abstract])) OR ("Heart attack*"[Title/Abstract])) OR ("Cardiac Infarc*"[Title/Abstract])) OR ("Cardiac Arrest*"[Title/Abstract])) OR (STEMI[Title/Abstract])) OR (NSTEMI[Title/Abstract])) OR ("Myocardial Ischemia"[Title/Abstract])) OR ("Cardiovascular Stroke*"[Title/Abstract])) OR ("Cardiac Stroke*"[Title/Abstract])  (((((“Major adverse cardiovascular event*”[Title/Abstract]) OR ("cardiovascular event*"[Title/Abstract])) OR ("cardiac event*"[Title/Abstract])) OR ("cardiovascular death*"[Title/Abstract])) OR ("cardiac death*"[Title/Abstract])) OR (“Cardiac revascularization”[Title/Abstract]) | 1,675 |
| Scopus | TITLE-ABS-KEY ("Major adverse cardiovascular event*") OR TITLE-ABS-KEY ("cardiovascular event*") OR TITLE-ABS-KEY ("cardiac event*") OR TITLE-ABS-KEY (MACE) OR TITLE-ABS-KEY ("cardiovascular death*") OR TITLE-ABS-KEY ("cardiac death*") OR TITLE-ABS-KEY ("Cardiac revascularization")  TITLE-ABS-KEY ( “adrenergic beta antagonist” ) OR TITLE-ABS-KEY ( “beta blocker*” ) OR TITLE-ABS-KEY ( “b-blocker*” ) OR TITLE-ABS-KEY ( “beta antagonist*” ) OR TITLE-ABS-KEY ( “beta adrenoreceptor antagonist” ) OR TITLE-ABS-KEY ( “beta adrenergic receptor antagonist” ) OR TITLE-ABS-KEY ( “beta adrenergic blocking agent” ) OR TITLE-ABS-KEY ( “adrenergic beta-1 receptor antagonists” ) OR TITLE-ABS-KEY ( acebutolol ) OR TITLE-ABS-KEY ( alprenolol ) OR TITLE-ABS-KEY ( atenolol ) OR TITLE-ABS-KEY ( betaxolol ) OR TITLE-ABS-KEY ( bisoprolol ) OR TITLE-ABS-KEY ( bunolol ) OR TITLE-ABS-KEY ( bupranolol ) OR TITLE-ABS-KEY ( Bucindolol ) OR TITLE-ABS-KEY ( carteolol ) OR TITLE-ABS-KEY ( celiprolol ) OR TITLE-ABS-KEY ( Carvedilol ) OR TITLE-ABS-KEY ( dihydroalprenolol ) OR TITLE-ABS-KEY ( esmolol ) OR TITLE-ABS-KEY ( iodocyanopindolol ) OR TITLE-ABS-KEY ( labetalol ) OR TITLE-ABS-KEY ( levobunolol ) OR TITLE-ABS-KEY ( metipranolol ) OR TITLE-ABS-KEY ( metoprolol ) OR TITLE-ABS-KEY ( nadolol ) OR TITLE-ABS-KEY ( Nebivolol ) OR TITLE-ABS-KEY ( oxprenolol ) OR TITLE-ABS-KEY ( penbutolol ) OR TITLE-ABS-KEY ( practolol ) OR TITLE-ABS-KEY ( pindolol ) OR TITLE-ABS-KEY ( propranolol ) OR TITLE-ABS-KEY ( sotalol ) OR TITLE-ABS-KEY ( timolol )  TITLE-ABS-KEY ( “Myocardial infarc*” ) OR TITLE-ABS-KEY ( “Heart attack*” ) OR TITLE-ABS-KEY ( “Cardiac Infarc*” ) OR TITLE-ABS-KEY ( “Cardiac Arrest*” ) OR TITLE-ABS-KEY ( STEMI ) OR TITLE-ABS-KEY ( NSTEMI ) OR TITLE-ABS-KEY ( “Myocardial Ischemia” ) OR TITLE-ABS-KEY ( “Cardiovascular Stroke*” ) OR TITLE-ABS-KEY ( “Cardic Stroke*” ) | 2,968 |
| Web of sciences | TS=(“adrenergic beta antagonist” OR “beta blocker*” OR “b-blocker*” OR “beta antagonist*” OR “beta adrenoreceptor antagonist” OR “beta adrenergic receptor antagonist” OR “beta adrenergic blocking agent” OR “adrenergic beta-1 receptor antagonists” OR acebutolol OR alprenolol OR atenolol OR betaxolol OR bisoprolol OR bunolol OR bupranolol OR Bucindolol OR carteolol OR celiprolol OR Carvedilol OR dihydroalprenolol OR esmolol OR iodocyanopindolol OR labetalol OR levobunolol OR metipranolol OR metoprolol OR nadolol OR Nebivolol OR oxprenolol OR penbutolol OR practolol OR pindolol OR propranolol OR sotalol OR timolol)  TS=(“Myocardial infarc*” OR “Heart attack*” OR “Cardiac Infarc*” OR “Cardiac Arrest*” OR STEMI OR NSTEMI OR “Myocardial Ischemia” OR “Cardiovascular Stroke*” OR “Cardic Stroke*”)  TS=(“Major adverse cardiovascular event*” OR “cardiovascular event*” OR “cardiac event*” OR MACE OR “cardiovascular death*” OR “cardiac death*” OR “Cardiac revascularization”) | 1,835 |
| Cochrane central | (adrenergic beta antagonist):ti,ab,kw OR (beta blocker*):ti,ab,kw OR (b-blocker*):ti,ab,kw OR (beta antagonist*):ti,ab,kw OR (beta adrenoreceptor antagonist):ti,ab,kw OR (beta adrenergic receptor antagonist):ti,ab,kw OR (beta adrenergic blocking agent):ti,ab,kw OR (adrenergic beta-1 receptor antagonists):ti,ab,kw OR (acebutolol):ti,ab,kw OR (alprenolol):ti,ab,kw OR (atenolol):ti,ab,kw OR (betaxolol):ti,ab,kw OR (bisoprolol):ti,ab,kw OR (bunolol):ti,ab,kw OR (bupranolol):ti,ab,kw OR (Bucindolol):ti,ab,kw OR (carteolol):ti,ab,kw OR (celiprolol):ti,ab,kw OR (Carvedilol):ti,ab,kw OR (dihydroalprenolol):ti,ab,kw OR (esmolol):ti,ab,kw OR (iodocyanopindolol):ti,ab,kw OR (labetalol):ti,ab,kw OR (levobunolol):ti,ab,kw OR (metipranolol):ti,ab,kw OR (metoprolol):ti,ab,kw OR (nadolol):ti,ab,kw OR (Nebivolol):ti,ab,kw OR (oxprenolol):ti,ab,kw OR (penbutolol):ti,ab,kw OR (practolol):ti,ab,kw OR (pindolol):ti,ab,kw OR (propranolol):ti,ab,kw OR (sotalol):ti,ab,kw OR (timolol):ti,ab,kw  (“Myocardial infarc*”):ti,ab,kw OR (“Heart attack*”):ti,ab,kw OR (“Cardiac Infarc*”):ti,ab,kw OR (“Cardiac Arrest*”):ti,ab,kw OR (STEMI):ti,ab,kw OR (NSTEMI):ti,ab,kw OR (“Myocardial Ischemia”):ti,ab,kw OR (“Cardiovascular Stroke*”):ti,ab,kw OR (“Cardic Stroke*”):ti,ab,kw  ("Major adverse cardiovascular event*":ti,ab,kw OR "cardiovascular event*":ti,ab,kw OR "cardiac event*":ti,ab,kw OR MACE:ti,ab,kw OR "cardiovascular death*":ti,ab,kw OR "cardiac death*":ti,ab,kw OR "Cardiac revascularization":ti,ab,kw) | 127 |

**Supplementary Table 2. Sensitivity analyses comparing pooled estimates from all studies with those excluding studies at moderate risk of bias across LVEF strata.**

| **Outcome** | **All studies** | | | **Without moderate bias studies** | | |
| --- | --- | --- | --- | --- | --- | --- |
|  | **N** | **HR** | **95% CI** | **N** | **HR** | **95% CI** |
| **MACE** |  |  |  |  |  |  |
| Not Reduced EF | 17 | 0.94 | 0.81 to 1.09 | 17 | 0.94 | 0.81 to 1.09 |
| EF: ≥ 40 | 8 | 0.99 | 0.76 to 1.29 | 8 | 0.99 | 0.76 to 1.29 |
| EF: 40-49 | **3** | **0.75** | **0.59 to 0.95** | **3** | **0.75** | **0.59 to 0.95** |
| EF: ≥ 50 | 6 | 0.99 | 0.81 to 1.20 | 6 | 0.99 | 0.81 to 1.20 |
| **All-cause mortality** |  |  |  |  |  |  |
| Not Reduced EF | 16 | 0.86 | 0.74 to 1.00 | **14** | **0.81** | **0.67 to 0.98** |
| EF: ≥ 40 | 8 | 0.87 | 0.71 to 1.07 | 8 | 0.87 | 0.71 to 1.07 |
| EF: 40-49 | 1 | 0.77 | 0.48 to 1.23 | 1 | 0.77 | 0.48 to 1.23 |
| EF: ≥ 50 | 7 | 0.86 | 0.66 to 1.13 | **5** | **0.77** | **0.44 to 1.32** |
| **Cardiovascular mortality** |  |  |  |  |  |  |
| Not Reduced EF | 15 | 0.86 | 0.74 to 1.00 | **12** | **0.77** | **0.59 to 1.02** |
| EF: ≥ 40 | 6 | 0.9 | 0.66 to 1.22 | 6 | 0.9 | 0.66 to 1.22 |
| EF: 40-49 | 2 | 0.73 | 0.53 to 1.03 | **1** | **0.69** | **0.39 to 1.21** |
| EF: ≥ 50 | 7 | 0.79 | 0.56 to 1.11 | **5** | **0.68** | **0.37 to 1.26** |
| **Re-MI** |  |  |  |  |  |  |
| Not Reduced EF | 9 | 0.86 | 0.64 to 1.14 | 9 | 0.86 | 0.64 to 1.14 |
| EF: ≥ 40 | 4 | 0.93 | 0.56 to 1.55 | 4 | 0.93 | 0.56 to 1.55 |
| EF: 40-49 | **1** | **0.45** | **0.23 to 0.89** | **1** | **0.45** | **0.23 to 0.89** |
| EF: ≥ 50 | 4 | 0.89 | 0.66 to 1.19 | 4 | 0.89 | 0.66 to 1.19 |
| **Stroke** |  |  |  |  |  |  |
| Not Reduced EF | 4 | 0.87 | 0.43 to 1.76 | 4 | 0.87 | 0.43 to 1.76 |
| EF: 40-49 | 1 | 0.84 | 0.29 to 2.41 | 1 | 0.84 | 0.29 to 2.41 |
| Stroke - EF: ≥ 50 | 3 | 0.88 | 0.34 to 2.26 | 3 | 0.88 | 0.34 to 2.26 |
| **Revascularization** |  |  |  |  |  |  |
| Not Reduced EF | 5 | 0.91 | 0.73 to 1.14 | 5 | 0.91 | 0.73 to 1.14 |
| EF: ≥ 40 | 2 | 0.84 | 0.50 to 1.42 | 2 | 0.84 | 0.50 to 1.42 |
| EF: 40-49 | 1 | 0.63 | 0.39 to 1.01 | 1 | 0.63 | 0.39 to 1.01 |
| EF: ≥ 50 | 2 | 1.06 | 0.86 to 1.30 | 2 | 1.06 | 0.86 to 1.30 |
| **HHF** |  |  |  |  |  |  |
| Not Reduced EF | 4 | 1.29 | 0.85 to 1.95 | 4 | 1.29 | 0.85 to 1.95 |
| EF: ≥ 40 | 2 | 1.88 | 1.21 to 2.92 | 2 | 1.88 | 1.21 to 2.92 |
| EF: 40-49 | 1 | 1 | 0.55 to 1.82 | 1 | 1 | 0.55 to 1.82 |
| EF: ≥ 50 | 1 | 0.9 | 0.54 to 1.51 | 1 | 0.9 | 0.54 to 1.51 |

**Abbreviations:** EF, ejection fraction; HR, hazard ratio; CI, confidence interval; MACE, major adverse cardiovascular events; Re-MI, recurrent myocardial infarction; HHF, hospitalization for heart failure.

**Supplementary Table 3. Certainty of evidence assessed using the GRADE approach for primary and secondary outcomes.**

| **Outcome** | **No. of studies (RCTs / Obs)** | **Total number** | **Pooled estimate (95% CI)** | **Certainty of the evidence (GRADE)** | **Reasons for the rating** |
| --- | --- | --- | --- | --- | --- |
| All-cause mortality | 5 RCTs | 23,524 | RR 0.98 (0.87–1.11) | Moderate | Downgraded 1 level for imprecision (CI crosses null; relatively few events) |
| Major adverse cardiovascular events (MACE) | 4 RCTs | 17,885 | RR 0.94 (0.87–1.02) | Moderate | Downgraded 1 level for imprecision |
| Cardiovascular mortality | 4 RCTs | 17,950 | RR 1.04 (0.77–1.40) | Moderate | Downgraded 1 level for imprecision |
| Recurrent myocardial infarction (Re-MI) | 5 RCTs | 23,524 | RR 0.89 (0.78–1.01) | Moderate | Downgraded 1 level for imprecision |
| Hospitalization for heart failure | 5 RCTs | 23,524 | RR 0.85 (0.67–1.07) | Moderate | Downgraded 1 level for imprecision |
| Stroke | 5 RCTs | 23,524 | RR 1.14 (0.77–1.68) | Moderate | Downgraded 1 level for imprecision |
| MACE in LVEF 40–49% subgroup | 3 Observational | 21,696 | HR 0.75 (0.59–0.95) | Low | Started at Low (primarily observational data); downgraded 1 level for risk of bias (potential confounding in observational studies) and indirectness (lack of dedicated RCT subgroup in this meta-analysis for this narrow stratum) |

**Abbreviations:** RCT, randomized controlled trial; Obs, observational studies; RR, risk ratio; HR, hazard ratio; CI, confidence interval; MACE, major adverse cardiovascular events; LVEF, left ventricular ejection fraction; GRADE, Grading of Recommendations Assessment, Development and Evaluation.

# **Supplementary Figure 1. All-Cause Mortality in Observational Studies**


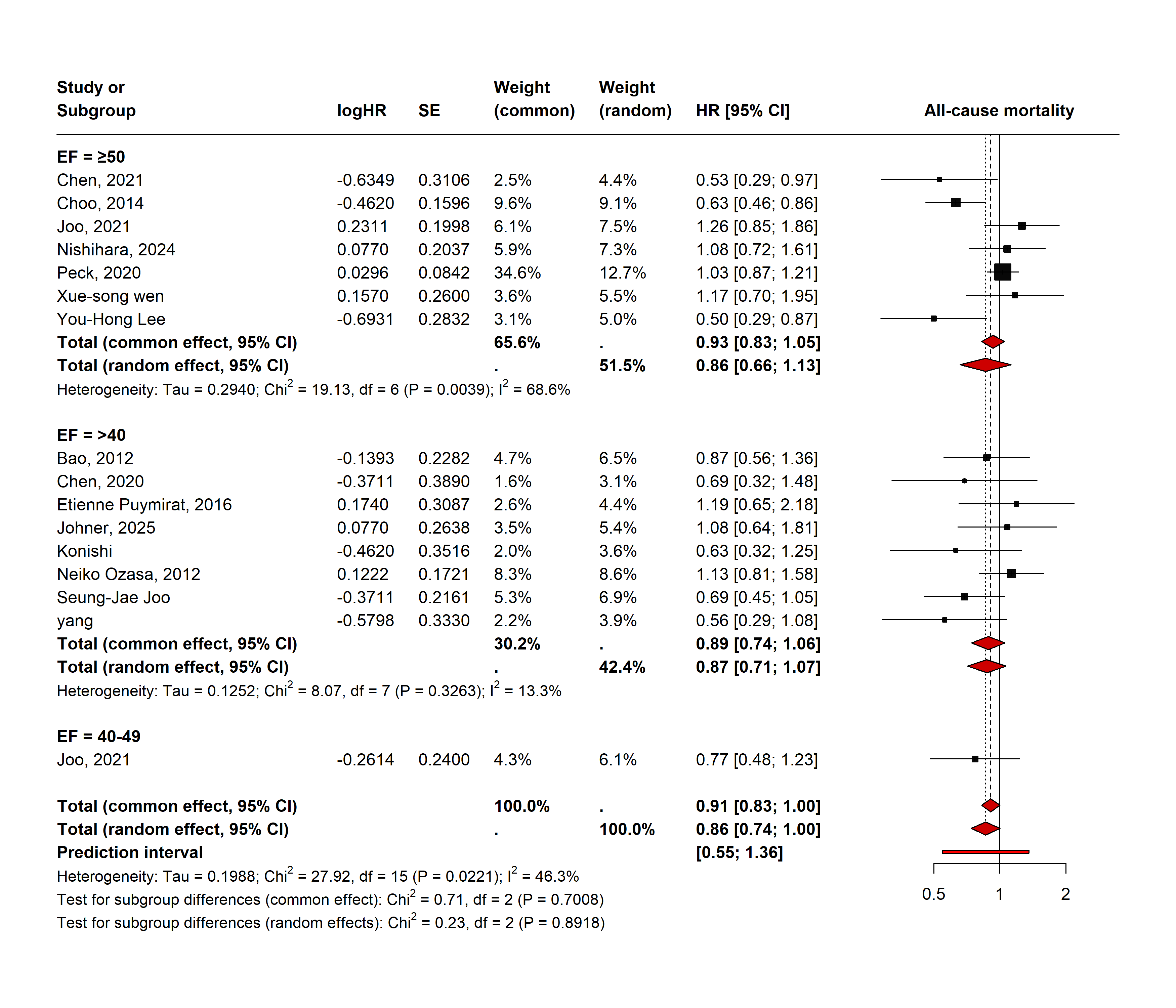


# **Supplementary Figure 2. MACE in Observational Studies**


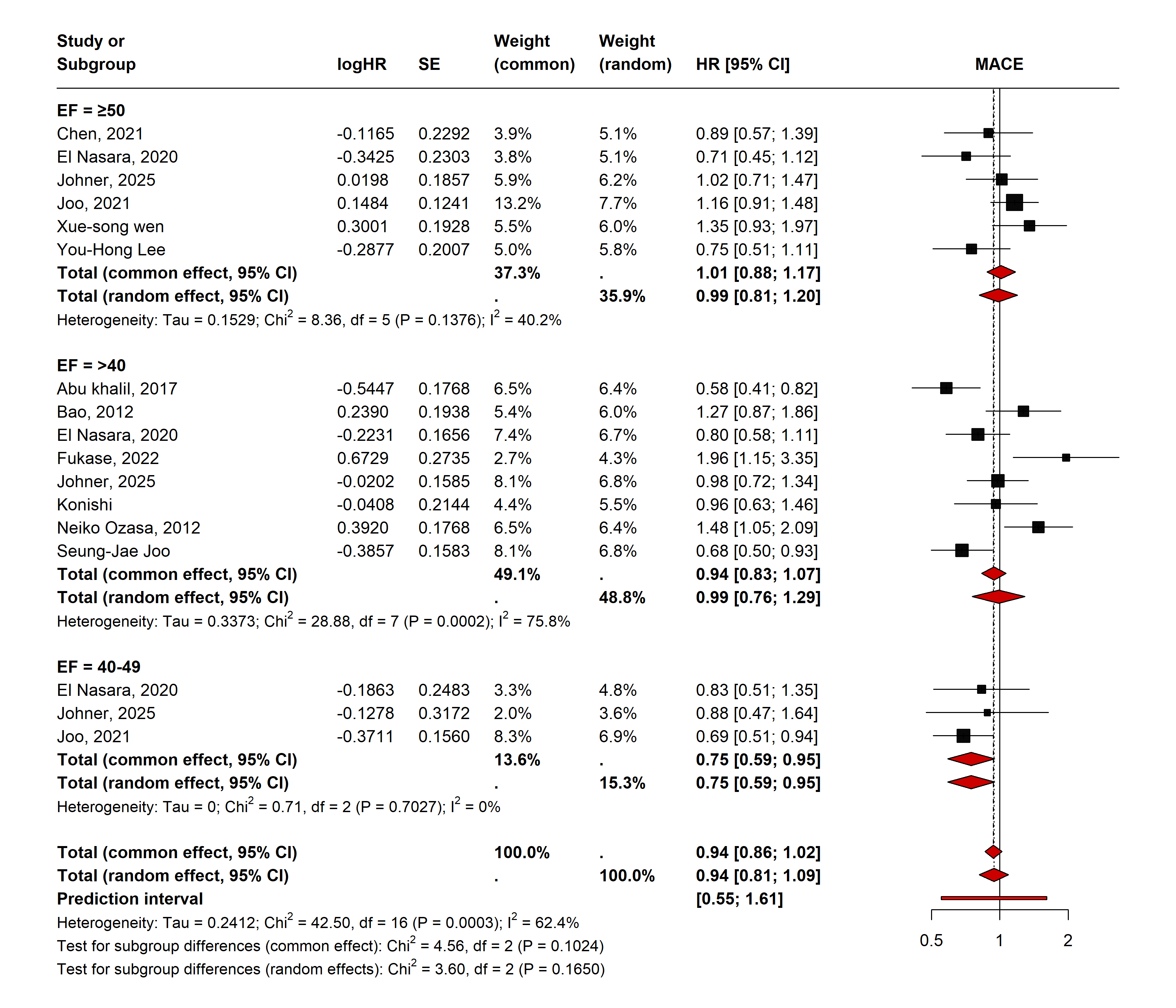


# **Supplementary Figure 3. Cardiac Mortality in Observational Studies**


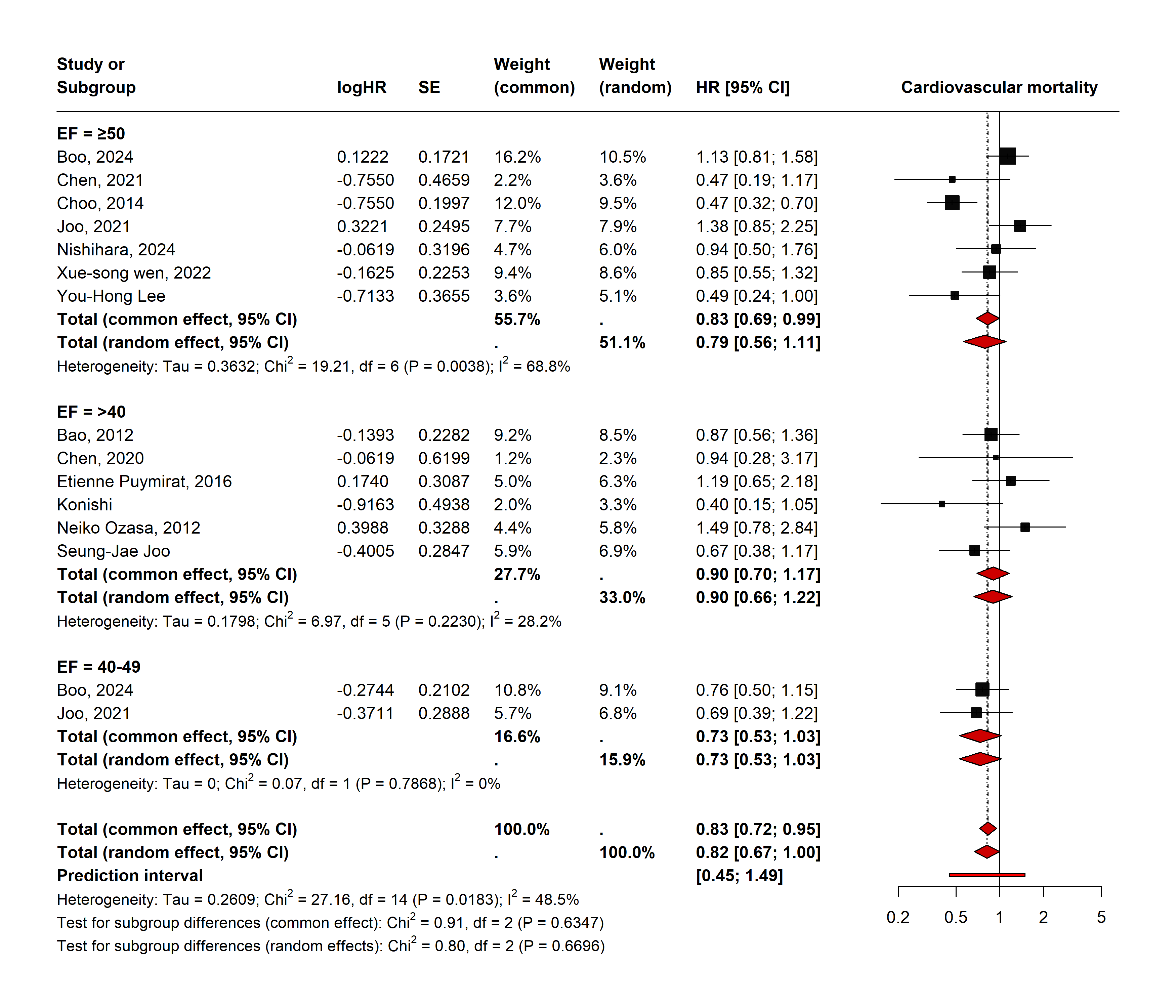


# **Supplementary Figure 4. Stroke in Observational Studies**


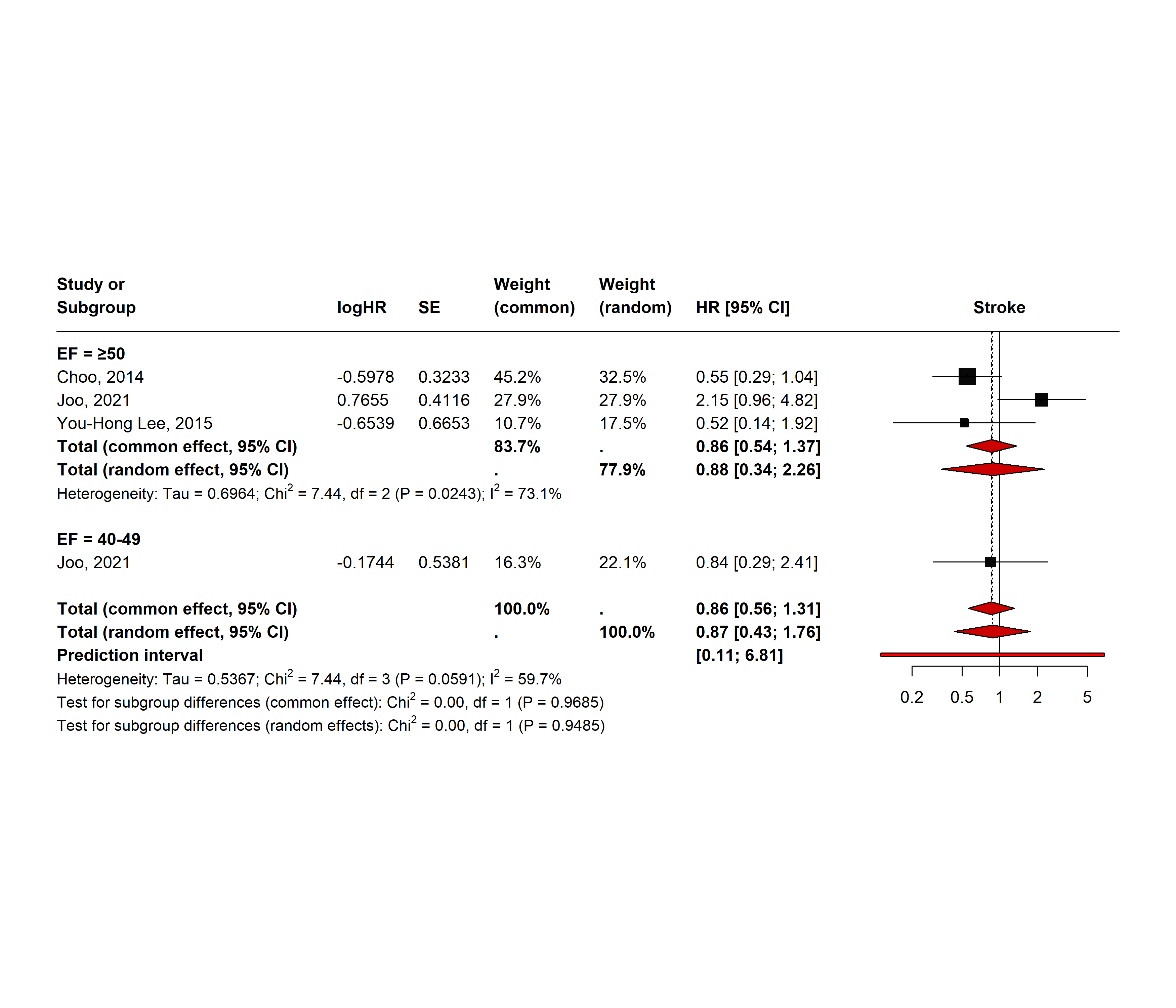


# **Supplementary Figure 5. Hospitalization for Heart Failure in Observational Studies**


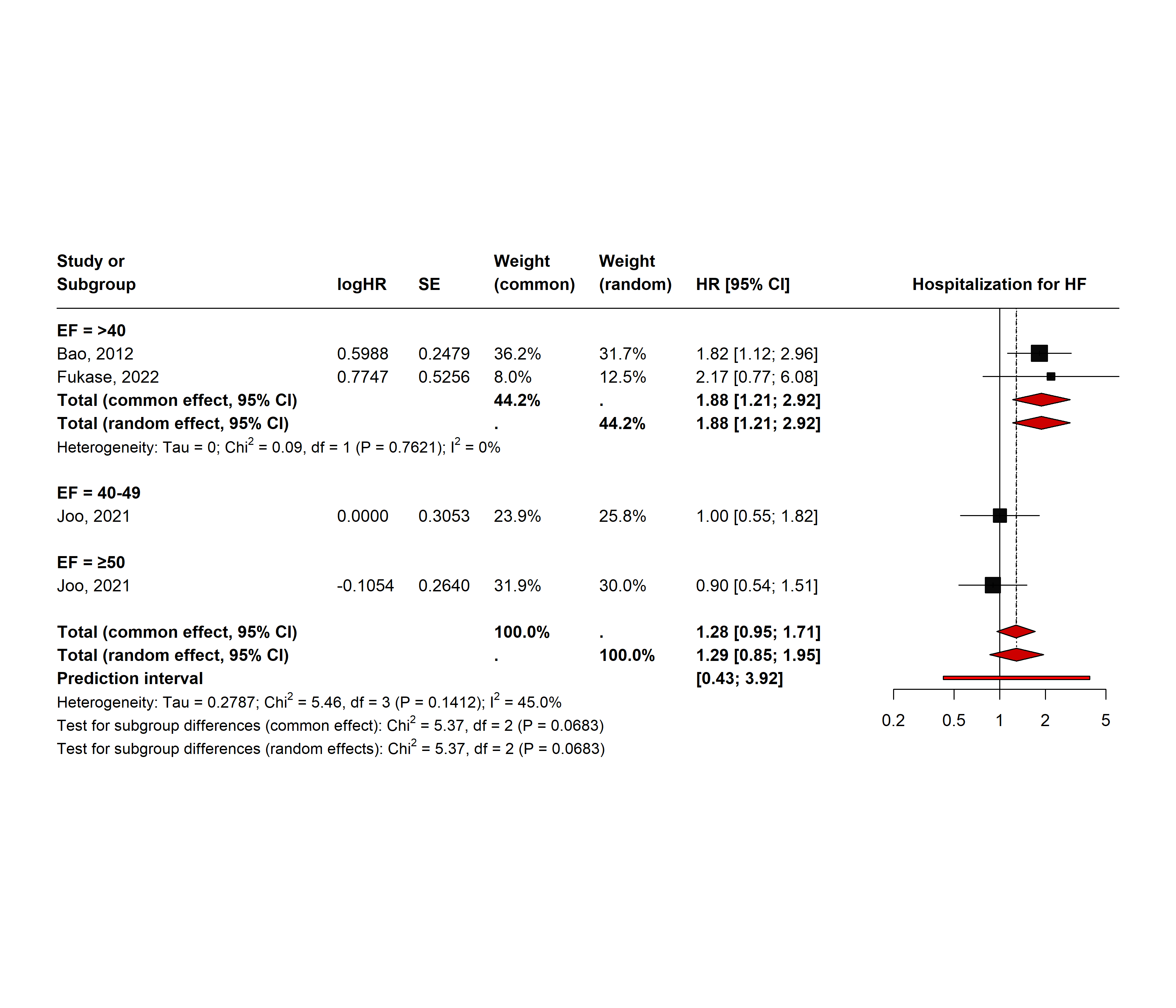


# **Supplementary Figure 6. Recurrent MI (Re-MI) in Observational Studies**


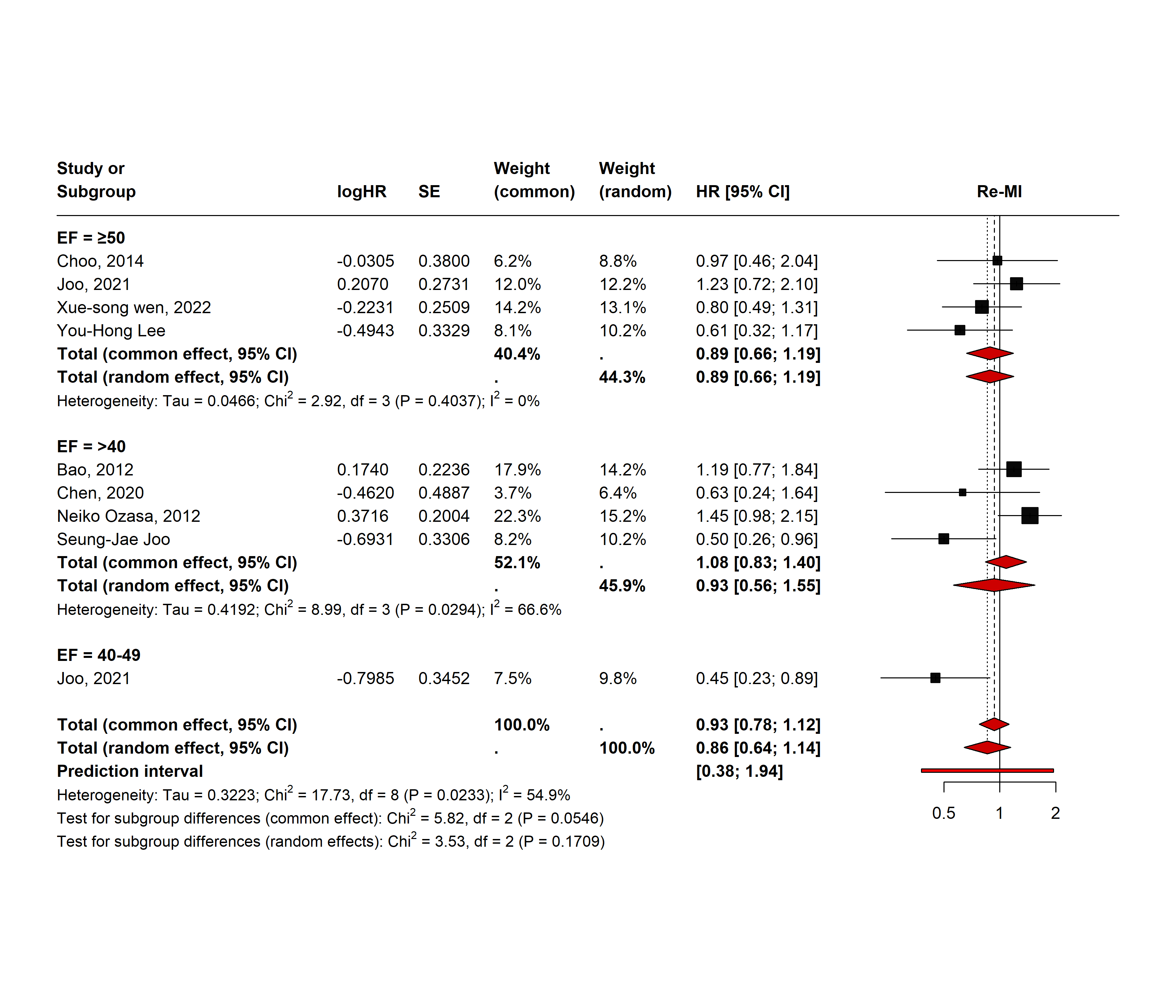


# **Supplementary Figure 7. Revascularization in Observational Studies**


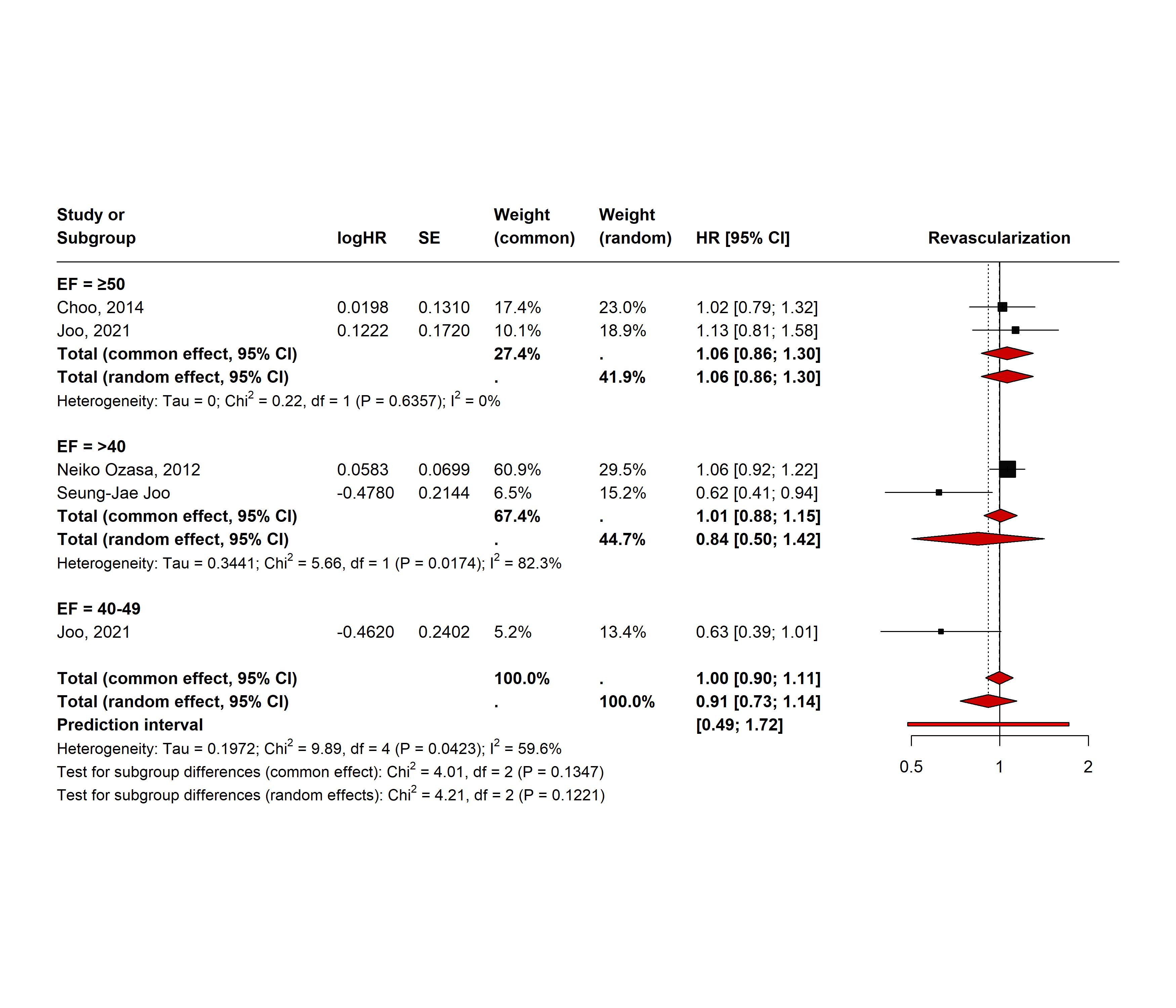

Supplement: Supplementary file 1 — Supporting Information Additional supporting information can be found online in the Supporting Information section. Table S1: Search strategy and search string details. Table S2: Sensitivity analysis. Table S3: Certainty of evidence. Figures S1, S2, S3, S4, S5, S6, and S7: Meta‐analysis results of observational studies. [file CDR-2026-2988999-s001.docx]
